# Concurrent Identification and Characterization of Protein Structure and Continuous Internal Dynamics with REDCRAFT

**DOI:** 10.3389/fmolb.2022.806584

**Published:** 2022-02-04

**Authors:** Hanin Omar, Aaron Hein, Casey A. Cole, Homayoun Valafar

**Affiliations:** Department of Computer Science and Engineering, University of South Carolina, Columbia, SC, United States

**Keywords:** REDCRAFT, RDC, protein, dynamics, computational, REDCAT, order tensor, PDBMine

## Abstract

Internal dynamics of proteins can play a critical role in the biological function of some proteins. Several well documented instances have been reported such as MBP, DHFR, hTS, DGCR8, and NSP1 of the SARS-CoV family of viruses. Despite the importance of internal dynamics of proteins, there currently are very few approaches that allow for meaningful separation of internal dynamics from structural aspects using experimental data. Here we present a computational approach named REDCRAFT that allows for concurrent characterization of protein structure and dynamics. Here, we have subjected DHFR (PDB-ID 1RX2), a 159-residue protein, to a fictitious, mixed mode model of internal dynamics. In this simulation, DHFR was segmented into 7 regions where 4 of the fragments were fixed with respect to each other, two regions underwent rigid-body dynamics, and one region experienced uncorrelated and melting event. The two dynamical and rigid-body segments experienced an average orientational modification of 7° and 12° respectively. Observable RDC data for backbone C′-N, N-H^N^, and C′-H^N^ were generated from 102 uniformly sampled frames that described the molecular trajectory. The structure calculation of DHFR with REDCRAFT by using traditional Ramachandran restraint produced a structure with 29 Å of structural difference measured over the backbone atoms (bb-rmsd) over the entire length of the protein and an average bb-rmsd of more than 4.7 Å over each of the dynamical fragments. The same exercise repeated with context-specific dihedral restraints generated by PDBMine produced a structure with bb-rmsd of 21 Å over the entire length of the protein but with bb-rmsd of less than 3 Å over each of the fragments. Finally, utilization of the Dynamic Profile generated by REDCRAFT allowed for the identification of different dynamical regions of the protein and the recovery of individual fragments with bb-rmsd of less than 1 Å. Following the recovery of the fragments, our assembly procedure of domains (larger segments consisting of multiple fragments with a common dynamical profile) correctly assembled the four fragments that are rigid with respect to each other, categorized the two domains that underwent rigid-body dynamics, and identified one dynamical region for which no conserved structure could be defined. In conclusion, our approach was successful in identifying the dynamical domains, recovery of structure where it is meaningful, and relative assembly of the domains when possible.

## Introduction

Mounting evidence demonstrates the importance of internal dynamics of biomolecules, including proteins, in their enzymatic and biological functions. A number of biologically important proteins have been the subjects of dynamic investigations, confirming the importance of internal dynamics in their function. The breathing motion of myoglobin (Shimada and Caughey, 1982; Cupane et al., 1988; Emerson et al., 1988; Bertini et al., 2003) can be cited as a historical instance of this property. Studies of other biologically important proteins such as lipases and hydrolases (Yu et al., 2016), dihydrofolate reductase (DHFR) (Bystroff and Kraut, 1991; Osborne et al., 2001), maltose binding protein (MBP) (Evenäs et al., 2001; Hwang et al., 2001; Millet et al., 2003; Tang et al., 2007), and others (Aramini et al., 2015; Kerns et al., 2015; Palmer, 2015; Wilson et al., 2015) have revealed the importance of internal dynamics in their function.

Computational approaches such as CHARMM (Brooks et al., 1983; Brooks et al., 2009), AMBER (Case et al., 2005; Salomon-Ferrer et al., 2013), GROMACS (Hess et al., 2008), or NAMD (Phillips et al., 2005) provide simulations of molecular dynamics (MD) from first principles. These platforms incorporate nearly all of the understood biophysical forces at the atomic level, and while the accuracy of the underlying potentials are not perfect, MD methods have the potential to generate reliable models of protein dynamics if given reasonably accurate starting points. X-ray crystallography is also used to study conformational sampling of some proteins (e.g., DHFR (Osborne et al., 2001), MBP (Diez et al., 2001; Duan et al., 2001)). Although studies of dynamics by X-ray crystallography can provide high-resolution descriptions of the multiple conformational states of proteins, these structures and/or their temporal occupancies may be perturbed by the crystal lattice. In fact, it is entirely plausible that functionally unimportant transient states are selected by a crystal lattice. In addition, the timescales of the dynamical events and occupancy of the conformational states are not recoverable by crystallography. Nuclear Magnetic Resonance (NMR) spectroscopy, including measurements of T_1_ and T_2_ relaxation rates (Barbato et al., 1992; Cavanagh et al., 2006; Lorieau et al., 2011), and relaxation-dispersion experiments (Lipari and Szabo, 1982), also provide powerful methods for investigating internal dynamics of macromolecules. However, there are few robust NMR studies of the equilibrium distributions of conformations that define the conformational landscape of the “native” protein structure.

Conceptually, from the experimental perspective it is difficult to separate the contribution of structure from dynamics since the two are intimately related. The existing approaches for characterization of protein dynamics from NMR measurements are typically performed in two separate steps—with the protein’s structure determined first, followed by an assessment of its motion using the calculated structure. Our recent work (Park et al., 2009; Shealy et al., 2010) has demonstrated the potential for obtaining erroneous structures when dynamically-averaged NMR data is best-fit to a single static structure. Subsequent mapping of dynamic information onto such an erroneous structure will likely lead to compromised models of motion. Therefore any attempt in structure elucidation that disregards the dynamics of a protein (or vice versa) can produce erroneous results (Tejero et al., 1996; Montelione et al., 2013). In this work, we demonstrate a more practical and rigorous approach to characterize a protein’s structure and its dynamics simultaneously through the use of Residual Dipolar Couplings (RDCs) (Tolman et al., 2001; Bryson et al., 2008; Park et al., 2009; Shealy et al., 2010; Valafar et al., 2012; Simin et al., 2014), which are sensitive reporters of both structure and dynamics (Tolman et al., 1997). The reported results will constitute the first instance of studying structure and dynamics of a protein from RDCs under a continuous and mixed-mode dynamics.

## Theoretical Background

### Residual Dipolar Couplings Data

Numerous reviews (Clore et al., 1998; Zhou et al., 1999; Prestegard et al., 2000; Tolman, 2001; Al-Hashimi et al., 2002a; De Alba and Tjandra, 2002; Blackledge, 2005) highlight the utility of RDC data in a broad spectrum of applications to biological macromolecules. RDCs have been used in studies of carbohydrates (Tian et al., 2001a; Azurmendi and Bush, 2002; Azurmendi et al., 2002; Adeyeye et al., 2003), nucleic acids (Al-Hashimi et al., 2000a; Tjandra et al., 2000; Vermeulen et al., 2000; Al-Hashimi et al., 2002a; Al-Hashimi et al., 2002b) and proteins (Cornilescu et al., 1999; Fowler et al., 2000; Andrec et al., 2001; Tian et al., 2001b; Clore and Bewley, 2002; Assfalg et al., 2003; Bertini et al., 2003). Until recently, the role of RDCs in structure determination has generally been to provide supplemental restraints to a large number of distance-based NOE restraints. Recent developments (Tian et al., 2001b; Dosset et al., 2001; Prestegard et al., 2005; Valafar et al., 2005; Simin et al., 2014) have demonstrated the success of structure determination of macromolecules by using primarily or exclusively RDC data. The use of RDCs can lead to a significant reduction in data collection and analysis (Raman et al., 2010; Shealy et al., 2010; Lange et al., 2012; Valafar et al., 2012; Tang et al., 2015) while providing simultaneous resonance assignment, structure determination, and identification of dynamical regions (Tian et al., 2001b; Bernadó and Blackledge, 2004; Prestegard et al., 2005; Valafar et al., 2005; Shealy et al., 2011; Cole et al., 2016).

RDCs arise from the interaction of two magnetically active nuclei in the presence of the external magnetic field of an NMR instrument (Prestegard et al., 2000; Clore et al., 1998; Tjandra et al., 1996; Tolman et al., 1995). This interaction is normally reduced to zero, due to the isotropic tumbling of molecules in their aqueous environment. The introduction of partial order to the molecular alignment reintroduces dipolar interactions by minutely limiting isotropic tumbling. This partial order can be introduced in numerous ways (Prestegard and Kishore, 2001), including inherent magnetic anisotropy susceptibility of molecules (Prestegard et al., 2000), incorporation of artificial tags (such as lanthanides) that exhibit magnetic anisotropy (Nitz et al., 2004), or in a liquid crystal aqueous solution (Prestegard and Kishore, 2001). The RDC interaction phenomenon can be formulated in different ways (Tolman et al., 1995; Bax and Tjandra, 1997). In our work we utilize the matrix formulation of this interaction as shown in Eq. 1. The entity *S* shown in Eqs 1, 2 represents the Saupe order tensor matrix (Prestegard et al., 2000; Valafar and Prestegard, 2004; Saupe and Englert, 1963) (the ‘order tensor’) that can be described as a 3 × 3 symmetric and traceless matrix. *D*_max_ in Eq. 1 is a nucleus-specific collection of constants, *r*_*ij*_ is the separation distance between the two interacting nuclei (in units of Å), and *v*_*ij*_ is the corresponding normalized internuclear vector. The order tensor formulation of the RDC interaction provides a convenient mechanism of probing internal dynamics of proteins. Decomposition of the alignment tensor (Losonczi et al., 1999; Valafar and Prestegard, 2004) can reveal information regarding the level of order (Pomeranz and Gershenfeld, 2000; Tolman et al., 2001; Valafar and Prestegard, 2004) and the preferred direction of alignment (Tolman et al., 2001; Valafar and Prestegard, 2004). A careful comparison of order tensors obtained from different regions of a macromolecule can provide a diagnostic tool in identifying relative orientations between structural elements and/or the presence of internal dynamics (Tolman et al., 2001; Valafar and Prestegard, 2004; Bryson et al., 2008).

$$D_{ij}=\left(\frac{D_{max}}{r_{ij}^{3}}\right)v_{ij}\text{∗}S\text{∗}v_{ij}^{T}$$ (1)

$$S=\left[\begin{array}{ccc} {\text{S}}_{\text{xx}} & {\text{S}}_{\text{xy}} & {\text{S}}_{\text{xz}}\\ {\text{S}}_{\text{xy}} & {\text{S}}_{\text{yy}} & {\text{S}}_{\text{yz}}\\ {\text{S}}_{\text{xz}} & {\text{S}}_{\text{yz}} & {\text{S}}_{\text{zz}} \end{array}\right],\;{\text{v}}_{\text{ij}}=\;\left(\begin{array}{c} \text{cos}\left({\text{θ}}_{\text{x}}\right)\\ \text{cos}\left({\text{θ}}_{\text{y}}\right)\\ \text{cos}\left({\text{θ}}_{\text{z}}\right) \end{array}\right)\;\;$$ (2)

The collection of RDC data imposes additional steps in sample preparation and data acquisition when compared to the requisites of the traditional data acquisition by NMR spectroscopy. Despite the additional requirements, the use of RDCs may be justified based on several of their unique features. Our most recent work (Peti et al., 2002) illustrated the sensitivity of NOEs and RDCs as reporters of protein structures. Based on this work, NOEs tend to lose sensitivity as the search approaches the native structure, while RDCs become more sensitive. Therefore, the addition of RDCs has the potential of improving the structural resolution of proteins studies by NMR spectroscopy. RDCs can also report molecular motions on time-scales ranging from picoseconds to microseconds (Tolman et al., 1997; Meiler et al., 2001; Peti et al., 2002), during which many functionally important events occur. Indeed, in the 10 ns–1 s timescale window, RDCs are the most sensitive of NMR parameters (Tolman et al., 2001). Therefore, in instances of investigating internal dynamics of macromolecules, the use of RDCs can be very beneficial if not necessary. In summary, RDCs have the unique property of simultaneously reporting structural and dynamics information, which has not been fully explored. In this work, we extend our previous work by presenting the first instance of simultaneous characterization of structure and dynamics that include continuous and mixed-mode internal dynamics.

### The Effect of Motion on Saupe Order Tensor

Previous works have described the theoretical aspects of the Suape Order Tensors (OTM) (Tolman et al., 1997; Shealy et al., 2011). Here we provide a more applied summary of this topic as it pertains to this report. Under purely theoretical and hypothetical conditions, a molecule that is absolutely devoid of any motion (internal or external tumbling) will achieve the highest level of order that is represented by the order tensor described in Eq. 3. Under realistic and unperturbed conditions, the isotropic tumbling of a macromolecule results in an order tensor that has been averaged to zero due to a uniform sampling of all possible molecular orientations. After inducing a tumbling anisotropy, a nonzero order tensor will be reintroduced based on the preferred orientation of the molecular tumbling, which is the origin of observing finite RDC data. In the absence of internal dynamics, the tumbling anisotropy is equally experienced by all portions of the molecule, and therefore OTMs reported by any portion of the molecule are equal to within the experimental error. The presence of internal dynamics will result in an OTM that is different than an OTM obtained from any other portion of the macromolecule. This is due to the fact that OTM from the dynamical region will consist of the effect of anisotropic molecular tumbling combined with the perturbation of internal dynamics. This is the primary principle that we employ in the development of our analysis. A systematic departure in OTMs reported from different portions of the protein are due to internal dynamics and can be used to identify dynamical regions, internally orchestrated motions, and be used in some instances to reconstruct the trajectory of motion (Cole et al., 2016).

$$S=\left[\begin{array}{ccc} -1/2 & 0 & 0\\ 0 & -1/2 & 0\\ 0 & 0 & 1 \end{array}\right]$$ (3)

## Materials and Methods

### Target Proteins

In this study we utilized dihydrofolate reductase enzyme (DHFR) that has been selected based on the substantial existing literature in support of major conformational changes when performing their enzymatic function (Bystroff and Kraut, 1991; Diez et al., 2001; Duan et al., 2001; Osborne et al., 2001).

Dihydrofolate reductase enzyme (DHFR) (Sawaya and Kraut, 1997) is a 159-residue long protein that has long been recognized for its central role in regulating tetrahydrofolate level in the cell, which directly aids in the synthesis of nucleic acid precursors. DHFR has been extensively studied and paramount evidence has confirmed its conformational changes as it binds to different intermediates (Fierke et al., 1987; Rod and Brooks, 2003; Antikainen et al., 2005; Mauldin and Lee, 2010). DHFR is a single-domain, monomeric molecule; the structure of which is divided into two subdomains: the adenosine binding subdomain and the loop subdomain. The gap separating the two subdomains is occupied by a nicotinamide ring, and the pteridine ring is located in the cleft between helices B and C. Four known states have been identified for this protein: open, closed, and occluded states depending on whether the active site is open, closed, or occluded by the loop. Due to internal dynamic, sometimes it becomes crystallographically unclear or invisible, hence the last state, known as the disordered state (Bystroff and Kraut, 1991). Although there exists ample evidence of the existence of internal dynamics, little is known regarding the exact nature of the structural rearrangement of this protein.

In this study we use DHFR to test the ability of our approach in concurrent characterization of structure and dynamics of proteins. To that end, we perform a fictitious, mixed-mode molecular dynamics simulation on DHFR (PDB-ID: 1RX2) in order to simulate RDC data and explore the possibility of identifying different dynamical regions of this protein by REDCRAFT, while providing atomic resolution structures for each dynamical domain. It is important to note that the imposed MDS is for illustration purposes only and it servers no useful information in recovering the actual dynamics of this protein in its native form.

### Molecular Dynamic Simulation

**FIGURE 1:**
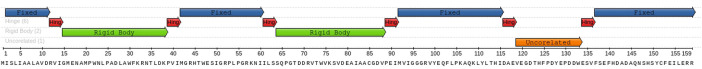
The regions of DHFR that were subjected to MD simulation.

**FIGURE 2:**
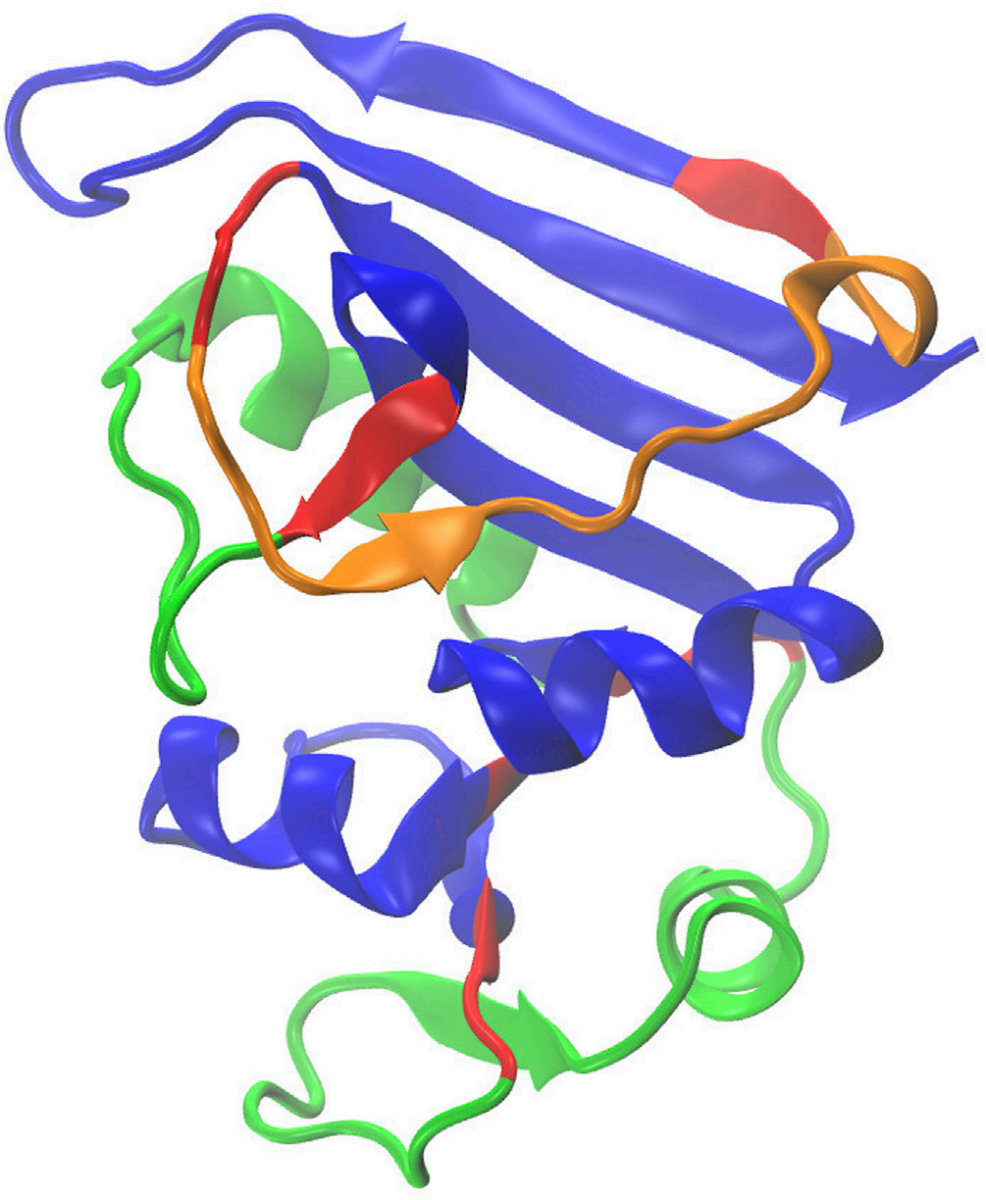
Structure of DHFR (PDB-ID 1RX2) that was used in this study with color annotation based on the simulated dynamics. The blue sections correspond to the fixed region while the green sections correspond to the rigid-body dynamics. The section illustrated in red section was subjected to no constraints and was subject to free motion (uncorrelated movement).

A fictitious, molecular dynamics simulation was implemented for DHFR based on some of the information available in the literature. More specifically, the structure PDB-ID 1RX2 was fractionated and subjected to various models of internal dynamics to better test our approach. The overall model of dynamics consisted of four fixed regions, two segments that underwent rigid-body dynamics, and one unstructured region. These segments were connected by hinge regions as shown in Figure 1 and Figure 2. As the first step in our MD simulation, the protein structure was minimized in order to arrive at a more equilibrated state. In the next step, A mixed-mode constrained molecular dynamics simulation was performed in XPLOR-NIH (Schwieters et al., 2003; Schwieters et al., 2006) (version 3.3) by keeping segments 1 (residue 1–11), 3 (residue42-60), 5 (residue 92–115), and 7 (residue 137–159) fixed in space. Segment 2 (residue 15–28) and segment 4 (residue 64–88) were constrained to experience rigid body dynamics by permitting the hinge regions (regions connecting each segment) to fluctuate freely in space. Segment 6 (residue 116–136) was allowed to freely move in space without any additional constraints and therefore experienced a melting of that domain. The simulation was conducted for 100,000 steps with step size of 0.0001 psec in a 2,000 K bath temperature. A total of 102 uniformly sampled frames were produced during the course of the molecular trajectory to be used during the calculation of ensemble RDC data.

### Calculation of RDC Data

Using the trajectory produced from the MD simulation, 102 frames were generated uniformly to span the entire course of the dynamics. Auxiliary tools were used to separate each of these frames in a PDB format and to generate a corresponding REDCAT file. The software package REDCAT (Valafar and Prestegard, 2004) was used to calculate the RDCs values for backbone C′-N, N-H^N^, and C′-H^N^ for each frame of the trajectory using the order tensors shown in Table 1 in two alignment media. REDCAT’s internal utility functions were used to create the observable RDCs by averaging the individual RDCs (for the three vectors) across the entire course of the dynamics (defined by 102 frames). To simulate a more realistic set of data, uniformly distributed noise in the range of ±0.5 Hz was added to all RDC data. These averaged RDCs were used for reconstruction of structure and study of the internal dynamics by REDCRAFT in a procedure highlighted in the following sections. It is important to comment on our choice of RDC data. Although a variety of highly informative RDC data (e.g., C_α_-H_α_, H_α_-H^N^, etc.) can be collected from smaller proteins, we have not used them in our studies since they may not be available in larger systems. To extend the applicable range of NMR spectroscopy to larger proteins, protons are exchanged with deuterons to improve spectral quality. Therefore in our study, we have confined the use of RDC data to what can be obtained from small or large and perdeuterated proteins. Finally, due to the existence of prolines, in general, the average number of RDCs is usually less than three per residue since only backbone N-H^N^ RDCs can be acquired. In the case of DHFR, the effective and average number of RDCs pre residue was reduced to 2.5 in each alignment medium.

**TABLE 1:** Order tensors used for RDC simulations.

	*S*_*xx*_	*S*_*yy*_	*S*_*zz*_	α	β	γ
*M*_*1*_	3 × 10^−4^	5 × 10^−4^	−8 x 10^−4^	0°	0°	0°
*M*_*2*_	−4 x 10^−4^	−6 x 10^−4^	10 × 10^−4^	40°	50°	-60°

### Context Specific Dihedral Constraints With PDBMine

PDBMine (Cole et al., 2019a) is a newly developed tool (https://ifestos.cse.sc.edu/PDBMine/) that performs an exhaustive search of the dihedral angles for a protein in the Protein Data Bank (Berman et al., 2000). As the first step, PDBMine creates a number of subsequences from the primary sequence of the query protein using a rolling window of size W. Therefore, for a protein of size N and a rolling window of size W, PDBMine creates N-W+1 subsequences. In the case of DHFR (159 residue protein) and a window size of 7, a total of 153 subsequences (residues 1–7, 2–8, 3–9 … 153–159) are created. As a second step, PDBMine gathers and aggregates an exhaustive list of all the observed dihedral angles for every residue in every subsequence present in the PDB. During the final step of its analysis, all the returned dihedral angles for all the subsequences are assembled into a final dihedral restraints for each residue of the query protein. In theory, a window size of one will reproduce the known Ramachandran dihedral space. Selection of a larger window size can be viewed as a context-sensitive Ramachandran space. Previous work (Cole et al., 2019b) has illustrated the differences between the dihedral spaces for a proline that precedes a glycine, versus a proline that succeeds a glycine. Therefore, having context specific estimations of dihedrals can be very useful in accelerating the task of structure determination. Another unique feature of PDBMine is its responsiveness; an exhaustive search of the PDB for a 159-residue protein will be completed in less than 10 min.

Under pragmatic conditions, use of the largest window size that produces a set of dihedrals is recommended. However, under testing conditions, it is important to exercise the necessary precautions to remove biases in the creation of the dihedral restraints. To that end, the primary objective is to avoid creation of the dihedral sets that are heavily populated with instance of 1RX2 or other homologous proteins. Therefore, any process that ensure diverse representation of dihedral angles will test the ability of REDCRAFT in identifying the correct dihedral angles among a large list of decoys. In this exercise, we explored window sizes of 3, 5, 7, and 9 after removing all instances of 1RX2 dihedrals. The window sizes of 3 and 5 produced an intractable number of hits, while the window size of 9 produced results that converged to the dihedrals of 1RX2 for some residues. The window size of 7 produced manageable results with at least 100 dihedrals that were separated from the actual dihedral of 1RX2 by more than 10° (some examples shown in the results section). REDCRAFT incorporates the results of PDBMine to improve its computation time by using the confined dihedral search space of the protein under investigation (in this case 1RX2). It is important to note that REDCRAFT can proceed in successful determination of protein structures in the absence of any dihedral constraints as demonstrated previously (Simin et al., 2014; Cole et al., 2021).

### Concurrent Study of Structure and Dynamics with REDCRAFT

During the past decade, several approaches and programs for structure determination from RDC data have been described (Saupe and Englert, 1963; Cornilescu et al., 1999; Clore and Bewley, 2002; Assfalg et al., 2003; Bernadó and Blackledge, 2004; Nitz et al., 2004; Bouvignies et al., 2005; Shealy et al., 2011). Each of these programs has different advantages and disadvantages. REDCRAFT (Clore and Schwieters, 2004; Bouvignies et al., 2005; Prestegard et al., 2005; Valafar et al., 2005; Shealy et al., 2010), sets itself apart from other existing software packages by deploying a more efficient and effective search mechanism. As a result, REDCRAFT can achieve the same structure determination outcome as other methods with less data (Cole et al., 2021). REDCRAFT also allows simultaneous study of structure and dynamics of proteins (Bryson et al., 2008; Simin et al., 2014; Cole et al., 2016). Applications of REDCRAFT in structure calculation have been demonstrated using aqueous (Bryson et al., 2008; Simin et al., 2014; Cole et al., 2015) and membrane (Shealy et al., 2010) proteins with as little as two RDCs per residue (Shen et al., 2009; Shealy et al., 2010; Shen and Bax, 2015) (in two alignment media).

REDCRAFT has introduced a novel approach to structure determination of proteins from RDC data (Cole et al., 2021). Aside from an unorthodox search method that is robust and fast (Cole et al., 2021), REDCRAFT employs an incremental strategy to structure determination in contrast to the all-at-once approach that is adopted by other existing methods. REDCRAFT’s incremental structure determination strategy has certain advantages and starts with a search for the optimal torsion angles that join two neighboring peptide planes. This seed dipeptide plane is recursively extended by one residue at a time by exploring a directed and extensive combinatorial search of the dihedral angles that extend the seed structure by one peptide plane (or amino acid) that optimally satisfies the RDC constraints. This process can start from the N-terminus of the protein and continue until the C-terminal end, or traverse the structure of the protein in the reverse order (C to N-terminus). The structural fitness that is produced by REDCRAFT during the course of fragment extension (from dipeptide to the entire protein) is termed the “Dynamic Profile” (or DP), which plays an instrumental role in a number of analyses including assessing the quality of the final structure or elucidation of internal dynamics. Using the Dynamic Profile, we have defined a process that allows for simultaneous identification and characterization of structure and internal dynamics. This process consists of three functional steps: standard structure determination, identification of internal dynamics (hinge regions), a grouping of the structural domains (coordinated dynamics), followed by reconstruction of the atomic resolution dynamics when possible. While the last step in the reconstruction of atomic-resolution of dynamics has been discussed in our previous work (Bryson et al., 2008; Shealy et al., 2010; Cole et al., 2015; Cole et al., 2021), the former steps have not been fully described in the literature. In addition, our previous work has been applied to the cases of finite and discrete state dynamics. In this work, we will define and test a more rigorous method of studying continuous and mixed mode dynamics. The four comprehensive steps are as follows:

#### Standard Structure Determination

Structure calculation of static proteins with REDCRAFT using RDC data has been well described (Cole et al., 2021). The DP of a static protein (or a static segment of a protein) generally starts with a low RDC fitness value due to the lack of experimental constraints. The underdetermined system generally produces a RDC fitness value of 0 and gradually increases during the elongation of the dipeptide seed. As the system becomes overdetermined, the RDC fitness reported by DP will increase to approximately the value of experimental error in data acquisition. Structural error defined by the actual deviation of peptide geometries from an ideal geometry (e.g., perfect planarity of the peptide planes, bond lengths, bond angles, etc.) is another source of error. Previous work has empirically determined this error to consist of 20% of the experimental data acquisition error (± 0.2 Hz in this case) (Cole et al., 2021). Supplementary Figure S1 presents an example of a typical DP for a static protein with the experimental error of ±1.0 Hz.

#### Identification of Hinge Regions and the Mode of Dynamics

The order tensor obtained from a dynamical portion of a protein will incorporate the effect of overall molecular tumbling and the effect of internal dynamics of that region. Therefore, order tensors reported from two domains of the same protein that undergo different regiments of dynamics will be incongruent. This difference in order tensors will be manifested as a sudden increase in the DP as REDCRAFT will be unable to identify a single order tensor and a static structure that will satisfy all the RDC constraints. Therefore, a sudden rise in the DP (as illustrated in the Supplementary Figure S2) that clearly exceed the expected error should be interpreted as the hinge region and signifies a transitional region between two distinctly different domains of the same protein. In such instances, the structure of the protein up to the onset of dynamics can be considered as an acceptable structure produced by REDCRAFT. To investigate the structure of the proceeding portion of the protein, a new structural fragment can be initiated a few residues past the hinge region. In our experiments, we use a skip region of 5 residues and repeat the step 1 above. If the new fragment exhibits a well-behaved DP, then the structure will be accepted as a rigid-body, otherwise, repeat the skip-ahead-region until a rigid-body is discovered. In this process any contiguous region that does not produce a well-behaved DP can be considered undergoing dynamics without any preserved structure, which we term uncorrelated dynamics. Our choice of the term “uncorrelated” is to denote any existing correction between the individual peptide planes of a fragment. Although in practice a gap size of one residue can be used to more accurately establish the hinge regions, a larger gap size is recommended in order to reduce the number of iterations that are needed to pass the hinge region. A more precise exploration of the hinge regions can be conducted at the later stages once the fragments are fully identified. At that point, each fragment can be extended on the C and N termini to more accurately identify the hinge regions.

#### Grouping of the Structural Domains

The next step in the process consists of assembling the individual fragments into larger domains based on their orchestrated internal dynamics. This process will allow the integration of fragments that are separated in the primary sequence but undergo a coordinated motion. The process of identifying the fragments that exhibit no relative internal motion with respect to each other will also complete the proper spatial orientation of the fragments with respect to each other. This process will also identify different regions of the protein that are experiencing different internal dynamics regiments. The assembly of fragments in space is previously described (Al-Hashimi et al., 2000b) and consists of first expressing all the fragments in a common frame (referred to as the Principal Alignment Frame, PAF) of the first alignment medium. RDC data are insensitive to inversion about each of the PAF and therefore four orientations of fragments with respect to each other are indistinguishable from each other. To eliminate the inversion degeneracy of structure assembly in one alignment medium (Al-Hashimi et al., 2000b), four alternative orientations of each fragment need to be explored from the perspective of the second alignment medium. The four orientations consist of each fragment as it appears and rotated by the 180° about each of the principal axes of the PAF (x, y, and z) for medium one. These four alternative orientations will be evaluated for fitness to the RDCs in the second alignment medium and the correct structure should exhibit the lowest score. In this exercise we use Q-factor (Cornilescu et al., 1998) as the measure of fitness that normalizes for the strength of alignment. After the completion of this step, all the fragments that belong to the same regiment of internal dynamics will be assembled with a low Q-score. The remaining fragments with clearly defined structure can be considered domains that undergo their unique rigid-body dynamics. Finally, any fragment with an incoherent structure is a domain that undergoes uncorrelated dynamics.

#### Reconstruction of Atomic-Resolution Trajectory of Dynamics

Presence of any form of internal dynamics will perturb the order tensor reported by that region of a molecule. In principle, perturbation of the order tensor can be used to recover an atomic-resolution trajectory of dynamics in some instances such as the case of discrete state dynamics. Our strategy in reconstruction of atomic resolution trajectory of dynamics has been previously discussed and therefore not presented in this report (Cole et al., 2016).

## Results and Discussion

### Dihedral Constraints for DHFR Using PDBMine

**FIGURE 3:**
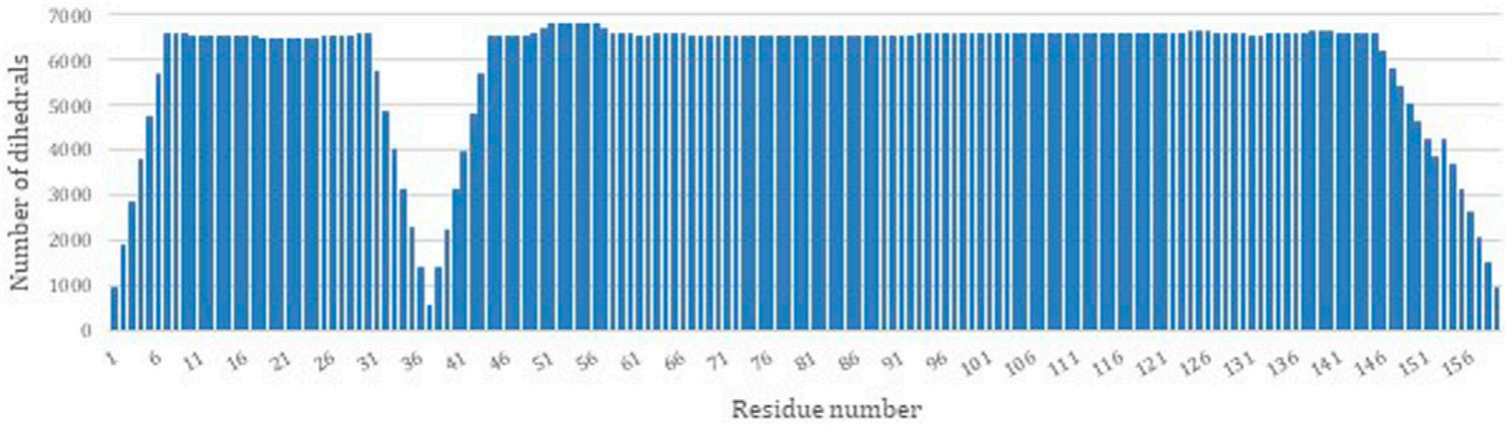
The number of dihedral angles returned by PDBMine using a window size of seven for the DHFR protein (PDB-ID 1RX2).

PDBMine was used as the first step to structure determination of DHFR by performing a search with a window size of 7. Figure 3 illustrates the number of hits that were identified by PDBMine with window size of 7 for each residue of DHFR. In average each residue received 5,923 possible dihedral angles with residues 37 and 57 receiving the least and the most (525 and 6,813 respectively) number of dihedral angles.

**FIGURE 4:**
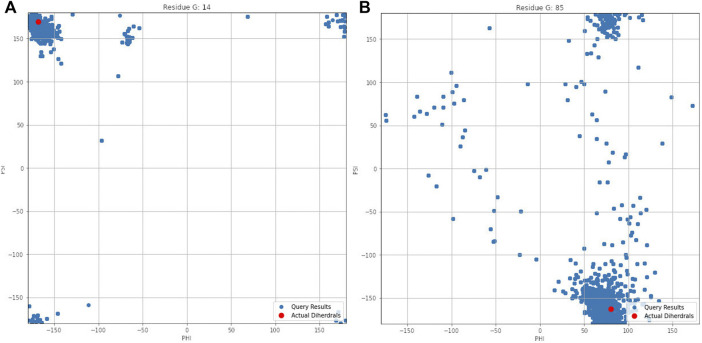
Dihedral angles produced by PDBMine using a window size of seven for residues **(A)** G14 and **(B)** G85 of DHFR protein.

Figure 4 illustrates the aggregated dihedral angles for residues G14 (panel A) and G85 (panel B). In this figure all the dihedral angles reported by PDBMine are illustrated in blue and the corresponding dihedral angles obtained from the PDB (1RX2) is illustrated in red. Several noteworthy observations can be stated. First, the results of PDBMine in principle converge to a Ramachandran space as a reducing window size. However, due to the context-specific nature of the search, a more restricted dihedral space is reported by PDBMine. The second notable observation further expands on the context specific nature of the PDBMine search and is illustrated in Figure 4. Both of the results correspond to a glycine, but they differ substantially due to the context in which the two glycines appear in the primary sequence. The third important point is to confirm the proper precautions that we have deployed to remove any unintended biases in our evaluations. It is clear from these figures that there are significant number of decoy dihedrals among which, REDCRAFT successfully selects the correct dihedral angle.

### Summary of MD Simulation

**FIGURE 5:**
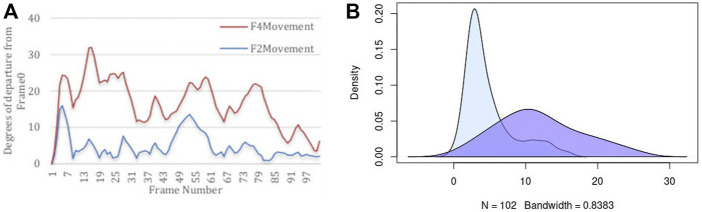
Descriptive statistics describing **(A)** the angular departure from the initial state (Frame0) for both Rigid-Body domains, and **(B)** the distribution of angular departure to assess the amount of time spent in each state.

It is important to quantify two aspects of internal dynamics. The first relates to capturing the magnitude of dynamics, and the second relates to the duration of time that was spent in different states. We first report the magnitude of dynamics for the rigid-body domains as an orientational departure from frame_0_ as the point of reference. Figure 5 illustrates the descriptive statistics regarding the movement of two Rigid-Body domains. Panel (A) of this figure displays the angular departure of each domain (F2 and F4) with respect to the fixed domains (F1, F3, F5, F7) measured between frame_i_ and frame_0_. Based on this information, Fragment 4 undergoes orientational rearrangement of as high as 32°, while Fragment 2 exhibits a much smaller motion of less than 15°. In addition to the magnitude of motion, it is important to assess the amount of time (or the number of frames) that each fragment spends in each orientational state during its trajectory. The frequency (or likelihood) of existing in a continuum of the orientational repositioning is illustrated in panel (B) of Figure 5. Based on this information, Fragment 2 spends a very small portion of its trajectory away from frame_0_, while spending most of the trajectory in the vicinity of the original state (less than 5°). Fragment 4 on the other hand, spends more than 50% of the time in an orientation more than 10° away from the original state. The general summary is that Fragment 2 undergoes small amount of structural rearrangement, while Fragment 4 exhibits a larger motion with respect to the fixed domains of the protein. It is important to state that the MD simulation of DHFR is purely engineered with the primary intention of exploring the sensitivity of our approach in detection of motion.

### Structure Determination of DHFR

As the first logical step, the structure of DHFR was determined in its entirety using REDCRAFT using Ramachandran dihedral restraints. As expected, this attempt at structure determination produced unsatisfactory results as indicated by the unacceptable fitness to the RDC data (1.14 Hz), and therefore are succinctly summarized here. The additional details are provided in Supplementary material in Supplementary Table S1 and Supplementary Figure S3. In summary, the overall structure exhibited 29 Å of bb-rmsd with respect to 1RX2 over the entire length of the protein with a fitness score of 1.14 Hz to the RDC data. The bb-rmsd computed over each of the fragments exhibited an average of 4.8 Å with localized similarities ranging from 0.8 to 9.7 Å.

**FIGURE 6:**
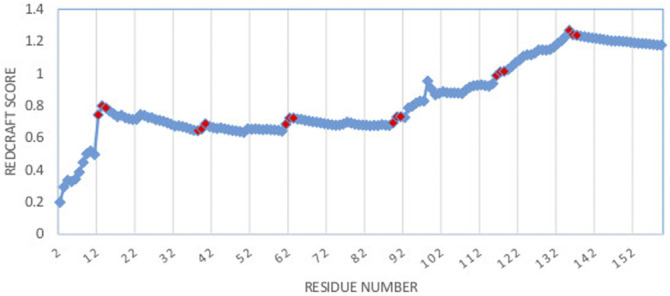
Dynamic profile of REDCRAFT for DHFR from residue 1 to 159. Hinge regions from the implemented MD simulation and marked in red to illustrate the correlation between the anomalous increases in DP and the transition between fragments with different internal dynamics.

As a more interesting case, the structure of DHFR was computed by REDCRAFT using the context specific dihedral restraints produced by PDBMine. The examination of the REDCRAFT’s DP will be crucial in assessing its success in the structure determination of this protein. The DP generated by REDCRAFT (shown in Figure 6) exhibits two indicators of the internal dynamics and therefore, a poor structure determination session. First, the final value of the fitness to the RDC data (1.2 Hz) compared to the expected value of 0.6 Hz (corresponding to the simulated error) indicates a failed attempt at structure determination. Second, the existence of sudden and anomalous increases in the DP in various places (e.g., at residues 12–14) is a potential indicator of internal dynamics that requires further examination. It is important to note the close correlation between the sudden increases in the DP and the location of hinge regions of our simulation (denoted by red markers in Figure 6).

**FIGURE 7:**
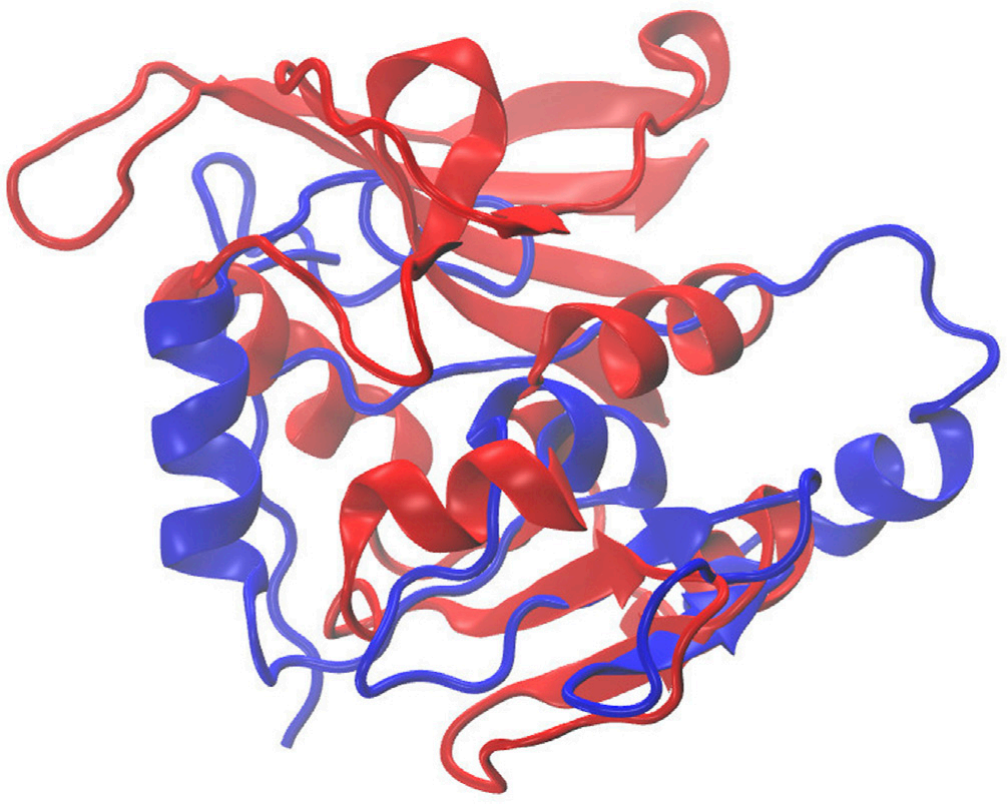
Superposition of the structure of 1RX2 (red) over the structure determined by REDCRAFT (blue). The two structures exhibit 21 Å of bb-rmsd.

Figure 7 illustrates the superimposed structure of DHFR (1RX2 shown in red) and the REDCRAFT recovered structure (shown in blue) by disregarding the existence of internal dynamics. Table 2 highlights the detailed results of comparing the structure of REDCRAFT to 1RX2. As a summary, the two structures exhibit a bb-rmsd of 21 Å and the comparison of fragments exhibit structural similarity in the range of 0.7 to 3 Å. Based on this information, in addition to the divergence in the overall structure, the structural error is also manifested in local fragments. It is important to note that the improved localized structural similarity is due to the effective restraining of the dihedrals accomplished by PDBMine. It is also important to note while the inclusion of PDBMine constraints improved the structural quality of our analysis, there is still substantial room for improvement.

**TABLE 2:** The BBRMSD of the different fragments generated through the complete run of REDCRAFT from residue 1 until residue159 of DHFR.

Fragment number	Residue range	BBRMSD with 1RX2
Whole protein	1–159	21 Å
Fragment 1	1–11	0.7 A
Fragment 2	16–38	0.73 A
Fragment 3	44–60	0.9 A
Fragment 4	64–88	2.2 A
Fragment 5	97–115	2.4 A
Fragment 6	116–137	??
Fragment 7	138–159	0.7 A

### Fragmented Structure Characterization

*Fragment 1: Residue 1–11*—In consideration of the results shown in the previous section, fragmented study of the protein was conducted. The results of REDCRAFT for the region consisting of residues 1–11 exhibits an acceptable fitness score (around 0.5 Hz), and is devoid of any sudden increase. Therefore, the structure is deemed acceptable as the first fragment of this protein. Implementing steps 1 and 2 listed in the Methods section, the fragmented study continues from residue 16 (after skipping ahead 5 residues).

**FIGURE 8:**
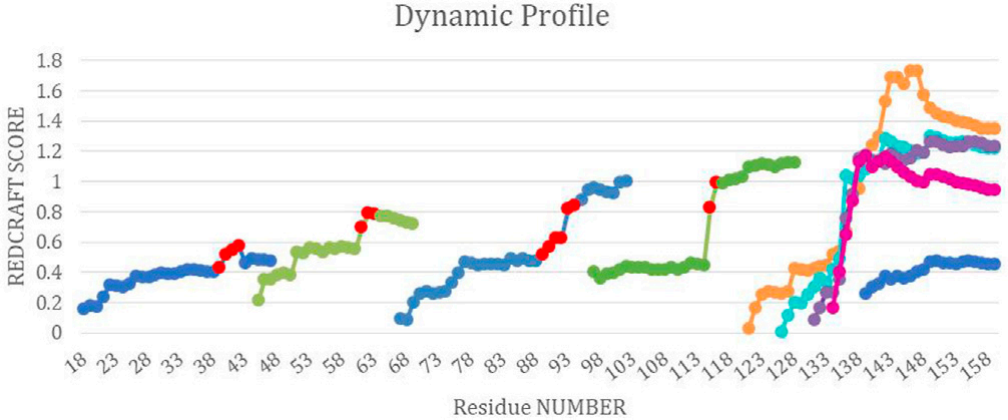
The combined dynamic profile for all REDCRAFT runs. The blue segments represent the dynamic profile of the fixed regions in DHFR, the green segments represent the dynamic profile for the rigid body dynamic parts of DHFR, different runs for the uncorrelated dynamics fragment are represented in orange, cyan, purple and pink. Last, the red points indicate the start of increase in scores in the specific dynamic profile for that run.

*Fragment 2: Residue 17–38*—Structure calculation of DHFR can proceed by investigating a new fragment. The start of the new fragment is based on skipping a fixed number of residues (i.e., 5 residues) from the onset of dynamics to pass the hinge region. The start of a new fragment essentially resets the calculation of an order tensor and therefore removes any inconsistency in the reported order tensors from two dynamically distinct domains of the protein. Therefore, structure calculation can proceed if a well-behaved DP is exhibited. Figure 8 illustrates the DP of the REDCRAFT for the new fragments starting at residue 17 and as expected, the REDCRAFT score increases at the beginning of the run due to lack of RDC data. Once stabilized, the general pattern is conserved until residue 38, at which point, the DP exhibits a distinct and anomalous increase in the REDCRAFT score. Indeed, residue 39 marks the beginning of the hinge regions and adjoins fragments 2 and 3 of this protein. Hence, we group residues 17–38 as the second Fragment in our investigation.

*Fragments 3, 4, 5, 6, and 7*—After completion of Fragment 2, a new structure calculation session was started from residue 44. As it can be observed in the DP for this segment (shown in Figure 8), the same general pattern as the previous two fragments is observed with an anomalous and notable increase in the REDCRAFT score at residue 61. This concluded the analysis of the third fragment that consisted of residues 44–60. The process of fragmented analysis was continued with the corresponding DP illustrated in Figure 8. The final completion of this process yielded four additional fragments F3 (44–60), F4 (65–88), F5 (97–116), and F7 (138–159). The range of the recovered fragments remarkably agree with the simulated MD. The DP of the only aberrant fragment, Fragment 6, is shown in Figure 8 as multiple attempts in structure recovery. Our first attempt at structure determination of this fragments started from residue 120 after skipping 5 residues from the end of the previous fragment. This attempt at structure determination was unsuccessful since the DP exhibited monotonically increasing score that exceeded the acceptable threshold of 0.6 Hz. The process of skipping forward by 5 residues was repeated with the objective of arriving at a well-behaved region of the protein. Each attempt at structure determination after skipping 5 residues is shown in Figure 8. This portion of the protein, unlike all other portions, never resulted in a well behaving DP due to the nature of its internal dynamics. Since the structure of this fragment was consistently modified in each frame, there is no conserved structure to recover, explaining the failure of structure calculation by REDCRAFT. This example also serves as a demonstration of cases where a gap region is larger than 5 residues.

**FIGURE 9:**
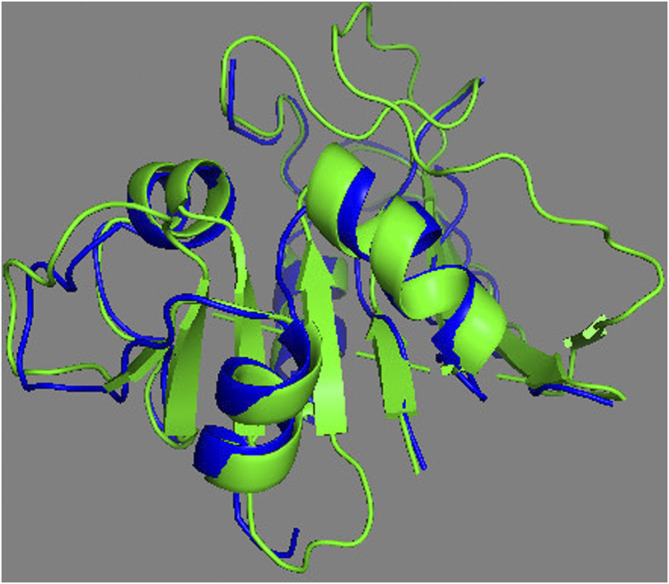
Superposition of the calculated fragments by REDCRAFT (blue) and the X-ray structure of DHFR (green).

The complete assessment of REDCRAFT’s results should consist of two parts. First, to evaluate the success of REDCRAFT in delineating different dynamical regions of the protein as described above. The second portion consist of assessing the structural accuracy of the recovered regions by REDCRAFT. Table 3 shows the results of the fragmented structure determination of DHFR by REDCRAFT while Figure 9 provides an illustration of the fragments (shown in blue) superposed on the corresponding regions of DHFR (shown in green). In Figure 9, we have omitted the REDCRAFT calculated structure of F6 due to the absence of a meaningful structure to compare. REDCRAFT was able to accurately recover the fragments of DHFR from three RDC data with an accuracy of less than 1 Å. It is important to note that these results are based on unrefined structures in order to expose and exhibit the raw capabilities of REDCRAFT. In practice however, these structure will benefit from refinement in platforms such as Xplor-NIH (Berman et al., 2000; Cole et al., 2019b), CNS (Brünger et al., 1998), or CYANA (Güntert and Downing, 2004) to name a few.

**TABLE 3:** The BBRMSD of the different fragments generated through the fragmented run of REDCRAFT.

Fragment #	Actual range	REDCRAFT range	BBRMSD with 1RX2
Fragment 1	1–11	1–11	0.5 Å
Fragment 2	15–38	16–38	0.65 Å
Fragment 3	42–60	44–60	0.71 Å
Fragment 4	64–88	64–88	1.2 Å
Fragment 5	92–115	97–115	0.75 Å
Fragment 6	116–137	116–137	N/A
Fragment 7	138–159	138–159	0.93 Å

*Fragment Assembly*—Following the structure determination of the individual fragments, the assembly process can proceed based on the procedure described in the Methods section. We start the assembly process by transforming all the fragments into their Principal Alignment Frame (denoted at PAF_1_) of the first medium and perform an initial investigation of their order tensor (OTM_1_). The OTM for each fragment in the second alignment medium is also established using the PAF_1_ as the common frame of comparison. Once the order tensors from all both alignment media have been canonicalized properly, a simple comparison of the order tensors will be sufficient to establish the relativly large motions between two fragments. In this case, F6 clearly was excluded based on the dissimilarity of its order tensors from the OTMs of any other fragment (due to one order of magnitude difference). However, since F2 and F4 were subjected to relatively small magnitudes of motion, the simple comparison of OTMs was inconclusive. A more sensitive discrimination of internal dynamics can be performed by assembling the fragments after examining all the inversion possibilities of each fragment. Table 4 provides a summary of the progressive fragment assembly using Q-Factor as a metric of fitness computed by REDCAT. The first column in this table indicates the progressively growing fragment during the course of the assembly. The nomenclature used in this column consists of the fragment number followed by subscript indicator of the fragment inversion examined in each evaluation. The second column indicates the fitness of the assembly to the combined RDC data in the first alignment medium. The following four columns signify the fitness of the assembly to the combined RDC data from the second alignment medium, after applying the indicated inversion to the last addition to the sequence. In these columns, *I, R*_*x*_ *, R*_*y*_, and *R*_*z*_ indicate no rotation (Identity or as is), rotation about *x, y*, and *z* axes respectively. The fragment assembly starts with the first fragment and as noted in the first row of this table. Note that there is no effect in the rotation of this fragment from the perspective of the second alignment medium. Using the first fragment in its original orientation, fragment 3 has been appended and Q-Factors have been computed for all of 4 possible orientations of F3 (not F1). Since the rotation about *y* yielded an acceptable score, its extension by the fragment 5 will be based on the y-rotated fragment 3. As an empirically accepted practice in the community, Q-Factor scores with values less than 0.2 reflect a high-quality structure and are deemed acceptable. (Cornilescu et al., 1998; Cole et al., 2021). Using this practice of evaluation, it is clear that fragments 1, 3, 5, and 7 can successfully be assembled as one unit (the fixed core), while fragments 2 and 4 cannot be successfully accepted as part of the fixed domain of the protein.

**TABLE 4:** Results of progressive fragment assembly as investigation all inversion degeneracies. The reported scores are Q-Factors determined by REDCAT.

Fragment #	*M1, I*	*I*	*R*_*x*_(*180˚)*	*R*_*y*_(*180˚)*	*R*_*z*_(*180˚)*
1	0.05	0.05	0.05	0.05	0.05
1_i_ 3	0.07	0.56	0.62	0.11	0.28
1 3_y_ 5	0.07	0.71	0.94	0.14	0.71
1 3 _y_ 5 _y_ 7	0.07	0.93	0.72	0.62	0.16
1 3 _y_ 5 _y_ 7_z_ 2	0.06	0.94	0.88	0.79	0.64
1 3 _y_ 5 _y_ 7_z_ 4	0.067	0.91	0.77	0.92	0.79

## Conclusion

Residual Dipolar Coupling are sensitive reporters of structure and dynamics covering a broad range of biologically relevant timescales. However, improper use of RDCs can lead to erroneous results, which may manifest as a faulty structure or an inaccurate model of dynamics. In fact, disregarding dynamics during the course of structure determination can be very detrimental as reported previously (Valafar et al., 2012). To fully extract the information reported by RDCs, it is imperative to utilize the appropriate analytic approach, in the appropriate manner. Here we have demonstrated that the use of REDCRAFT allows for clear identification of onset of internal dynamics in a protein. In the case of our simulated DHFR, each of the hinge regions was identified very accurately to within one or two residues. Proper isolation of fragments that exhibit a consistent internal dynamics regiment allows for the recovery of structural information after removing the influence of dynamics. In this study we have demonstrated the accurate recovery of structural fragments to within 1 Å of accuracy using only three RDC data acquired in two alignment media.

In addition to accurate structure determination, we demonstrated REDCRAFT’s ability to decipher between rigid-body and uncorrelated modes of dynamics as demonstrated with fragments 2, 4, and 6 of DHFR. Although the three domains underwent internal dynamics, REDCRAFT successfully recovered the structure of fragments 2 and 4, where structure was conserved during the course of the dynamics. On the other hand, the uncorrelated mode of dynamics does not present the conservation of structural coherence throughout the course of dynamics, which renders the exercise of structure determination moot. The nature of internal dynamics of different fragments was established during the course of the fragment assembly. In this step, fragments 1, 3, 5, and 7 were successfully assembled, affirming the fixed relationship between these fragments. The inability to assemble fragments 2 and 4 with the fixed core (fragments 1, 3, 5, and 7) of the protein, when combined with confidently computed structures concludes that the two domains undergo internal dynamics with respect to the core. In regard to the magnitude of dynamics, our previous work (Cole et al., 2021) related to discrete-state dynamics concluded the inability to identify dynamics with magnitude of less than 15° of movement. This observation was reconfirmed in this study as the distortion of DP in transition from the first fragment to the second was not as notable as the distortion of DP due to the larger dynamics of Fragment 4.

Finally, in our interpretation of DP distortions, we disregarded some anomalous increases in some instances. Except for Fragment 6, all other fragments exhibited such instances with the most notable ones appearing at residue 50 in Fragment 3 or residue 74 in Fragment 4. In such instances we have accepted the results since the net RDC-fitness remained within the experimental error. The origin of these subtle distortions is due to localized departure of peptide geometries from ideal geometries such as non-ideal omega angles, slightly modified bond angles, or bond lengths. These types of structural noise (Cole et al., 2021) are the basis of expanding the threshold of acceptable RDC-fitness by 20% of the experimental error and are easily rectified during the refinement process when peptide geometries are relaxed and allowed to deviate within an acceptable range (Cole et al., 2021).

## Data Availability

The original contributions presented in the study are included in the article/Supplementary Material, further inquiries can be directed to the corresponding author.
