# Supplementary figures and images for: Concurrent Identification and Characterization of Protein Structure and Continuous Internal Dynamics with REDCRAFT
Source: Front Mol Biosci. 2022 Feb 4;9:806584. doi: 10.3389/fmolb.2022.806584 (PMC8856112; doi:10.3389/fmolb.2022.806584)

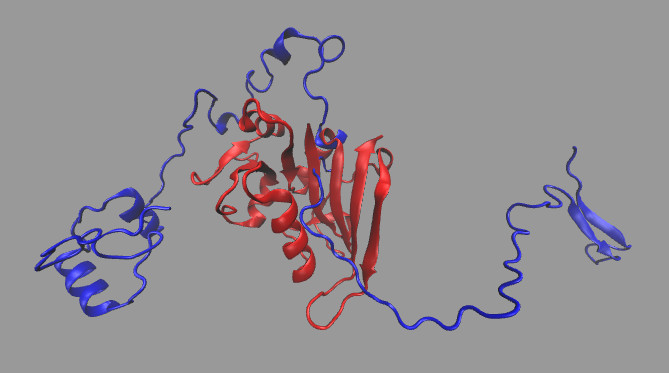

Supplement: Supplementary file 1 [file Image3.JPEG]

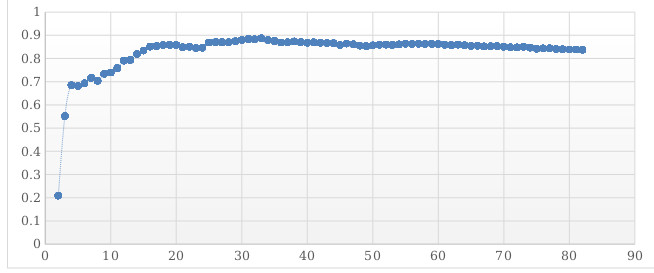

Supplement: Supplementary file 2 [file Image1.JPEG]

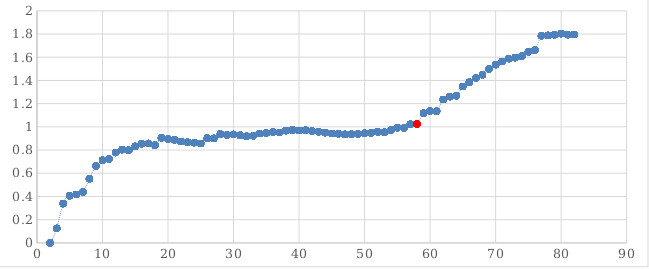

Supplement: Supplementary file 3 [file Image2.JPEG]
