# Supplementary material for: Concurrent Identification and Characterization of Protein Structure and Continuous Internal Dynamics with REDCRAFT
Source: Front Mol Biosci. 2022 Feb 4;9:806584. doi: 10.3389/fmolb.2022.806584 (PMC8856112; doi:10.3389/fmolb.2022.806584)
Supplement: Supplementary file 4 [file DataSheet1.docx]

Supplementary Material

# Supplementary Figures and Tables

## Supplementary Figures

Figure1: Typical DP in structure with no dynamics (generated from structure calculation of PDB id 1A1Z) with an experimental error of ±1Hz.


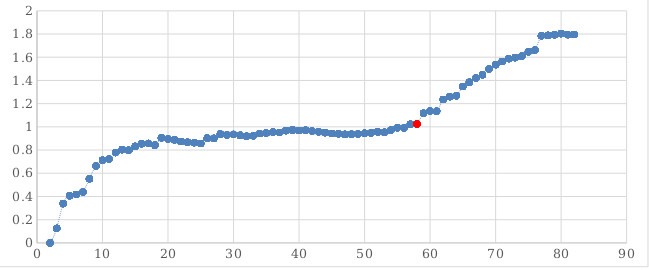
Figure 2: DP of PDB ID 1A1Z with a simulated 2 state motion starting at residue 58 (shown in red). A uniformly distribute noise of ±1Hz was added to all RDC data.


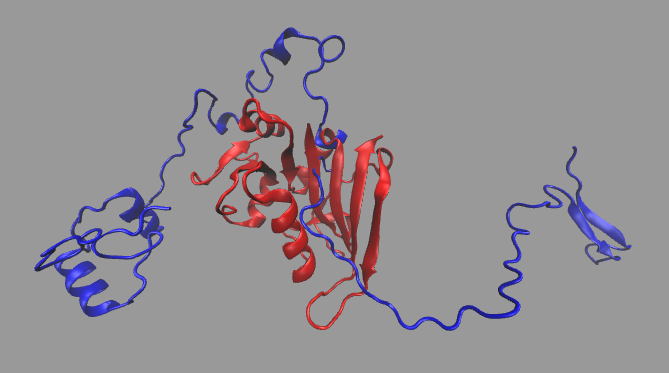
Figure 3: Structure of DHFR determined by REDCRAFT (shown in blue) using typical Ramachandron dihedral restraints superposed on the actual X-ray structure (shown in red) with more than 35Å of bb-rmsd.

## Supplementary Tables

Table 1: The structure computed by REDCRAFT using standard Ramachandron restraints. As expected, the structure is locally and globally compromised due to the influence of dynamics on RDC data.

| Fragment number | Residue Range | BBRMSD with 1RX2 |
| --- | --- | --- |
| Whole protein | 1 - 159 | >37Å |
| Fragment 1 | 1 - 11 | 2.0Å |
| Fragment 2 | 16 - 38 | 0.8Å |
| Fragment 3 | 44 - 60 | 5.5Å |
| Fragment 4 | 64 - 88 | 6.0Å |
| Fragment 5 | 93 - 114 | 9.7Å |
| Fragment 6 | 115-137 | 8.9Å |
| Fragment 7 | 138 - 159 | 0.7Å |
